# Exposure to Chlorpyrifos Alters Proliferation, Differentiation and Fatty Acid Uptake in 3T3-L1 Cells

**DOI:** 10.3390/ijms242216038

**Published:** 2023-11-07

**Authors:** Magdalena Czajka, Krzysztof Sawicki, Magdalena Matysiak-Kucharek, Marcin Kruszewski, Jacek Kurzepa, Paulina Wojtyła-Buciora, Lucyna Kapka-Skrzypczak

**Affiliations:** 1 Department of Molecular Biology and Translational Research, Institute of Rural Health, 20-090 Lublin, Poland; 2 Centre for Radiobiology and Biological Dosimetry, Institute of Nuclear Chemistry and Technology, 03-195 Warsaw, Poland; 3 Department of Medical Chemistry, Medical University of Lublin, 20-093 Lublin, Poland; 4 Department of Social Medicine and Public Health, Calisia University, 62-800 Kalisz, Poland; 5 World Institute for Family Health, Calisia University, 62-800 Kalisz, Poland

**Keywords:** adipogenesis, hypertrophy, hyperplasia, fatty acid uptake

## Abstract

Organophosphorus pesticides (OPs) are important factors in the etiology of many diseases, including obesity and type 2 diabetes mellitus. The aim of this study was to investigate the effect of a representative of OPs, chlorpyrifos (CPF), on viability, proliferation, differentiation, and fatty acid uptake in 3T3-L1 cells. The effect of CPF exposure on preadipocyte proliferation was examined by the MTT, NR, and BrdU assays. The impact of CPF exposure on the differentiation of preadipocytes into mature adipocytes was evaluated by Oil Red O staining and RT-qPCR. The effect of CPF on free fatty acid uptake in adipocytes was assessed with the fluorescent dye BODIPY. Our experiments demonstrated that exposure to CPF decreased the viability of 3T3-L1 cells; however, it was increased when the cells were exposed to low concentrations of the pesticide. Exposure to CPF inhibited the proliferation and differentiation of 3T3-L1 preadipocytes. CPF exposure resulted in decreased lipid accumulation, accompanied by down-regulation of the two key transcription factors in adipogenesis: C/EBPα and PPARγ. Exposure to CPF increased basal free fatty acid uptake in fully differentiated adipocytes but decreased this uptake when CPF was added during the differentiation process. Increased free fatty acid accumulation in fully differentiated adipocytes may suggest that CPF leads to adipocyte hypertrophy, one of the mechanisms leading to obesity, particularly in adults. It can therefore be concluded that CPF may disturb the activity of preadipocytes and adipocytes, although the role of this pesticide in the development of obesity requires further research.

## 1. Introduction

The World Health Organization defines overweight and obesity as abnormal or excessive fat accumulation that presents a risk to health [1]. Obesity itself has now become a metabolic disease of epidemic proportions that has numerous health consequences. Diabetes mellitus (particularly type 2; T2DM), a number of cancers, cardiovascular disease, hypertension, osteoarthritis, asthma, sleep apnea, thrombophlebitis, depression, sexual dysfunction (particularly in men), and easy fatigability are simply a few examples. Therefore, obesity represents a significant challenge for public health [2].

However, the etiology of T2DM and its connection with obesity is still not clearly defined. It is believed that T2DM results from environmental and lifestyle factors, such as poor diet, reduced physical activity, short or disturbed sleep, smoking, stress, depression, exposure to environmental chemicals [3], and obesity [4,5]. Recently, several studies have indicated that exposure to environmental pollutants may also be involved in the development of insulin resistance and T2DM. Among such environmental factors are pesticides, commonly used in different areas of our lives (e.g., agriculture, public health, households, and veterinary medicine). Consequently, pesticides, their metabolites and degradation products are detectable in the air, food, and water. In addition, workers involved in all stages of the production and application of pesticides are also at risk.

Organophosphorus pesticides (OPs) were extensively used in agriculture and horticulture; therefore, they are important factors in human environmental exposure. A representative example of OPs is chlorpyrifos (CPF). Although it is well established that OPs are neurotoxic due to the inhibition of acetylcholinesterase [6], CPF can also induce oxidative stress, disrupt the synthesis of macrobiomolecules, such as DNA, RNA, and proteins, affect the release and uptake of neurotransmitters, interact with various enzymes, disrupt neuronal differentiation, and affect signaling pathways [7,8]. Research indicates that CPF may be embryotoxic, teratogenic, or genotoxic; it may disrupt the immune system; and it may well be associated with gut microbiota dysbiosis [9,10,11,12,13,14,15]. It was also shown that CPF is hepatotoxic [9,16]. CPF can significantly increase the levels of serum AST, ALT, ALP, urea, and creatinine, which indicates liver and kidney damage [17,18]. Scientific research shows a correlation between occupational exposure to CPF and an increased risk of developing various types of cancer [19,20,21,22].

Recent studies indicate that pesticides, including CPF, may affect body weight, glucose, and lipid metabolism and induce hyperlipidemia, obesity, and T2DM. OPs and organochlorines are the most widely studied pesticides linked to such disturbances in humans and rodents [23,24]. It is suggested that pesticides may promote the commitment phase of adipogenesis, induce adipocyte differentiation, and affect metabolic homeostasis through PPARs. It has also been hypothesized that pesticides exert obesogenic effects, which are associated with sex steroid hormone dysregulation, affecting metabolic homeostasis through disturbing the thyroid hormone pathway, or affecting the gut microbiota [25]. There is increasing evidence obtained from in vitro animal studies and research in humans suggesting that exposure to pesticides can induce epigenetic changes [26]. The role of pesticide-induced epigenetic changes is also suggested in the development of human chronic diseases, including obesity; however, the exact mechanism of such an action has been insufficiently studied and the obtained results are inconclusive, thus further studies are still required [27,28].

Therefore, the aim of this study was to investigate the effect of CPF on the proliferation and differentiation of 3T3-L1 cells, as well as assess the effect of this pesticide on fatty acid uptake. 3T3-L1 cells are one of the most frequently used cellular in vitro models of preadipocytes for studying adipogenesis and the screening of obesogenic compounds. This cell line is also widely used to investigate cellular mechanisms associated with obesity, diabetes, and related disorders. Mature 3T3-L1 cells demonstrate most of the morphological and functional features that are typical of adipocytes in vivo [29,30,31,32,33]. The obtained results may be important in the context of environmental risk factors predisposing to obesity and T2DM.

## 2. Results

### 2.1. Effect of CPF on Viability and Proliferation of 3T3-L1 Cells In Vitro

In this study, we used three different “proliferation” assays to assess different cellular functions. MTT assay measures the activity of cellular NADH-dependent oxidoreductases, thus reflecting rather the extent of cellular metabolism than cell proliferation per se. Neutral red assay measures the integrity of cellular membranes; thus, it roughly corresponds to the number of life cells. Finally, BrdU assay measures the intensity of DNA synthesis, thus corresponding to the cell’s potential to proliferate. Taken together, these assays allow estimation of cellular proliferative and metabolic status.

**Figure 1:**
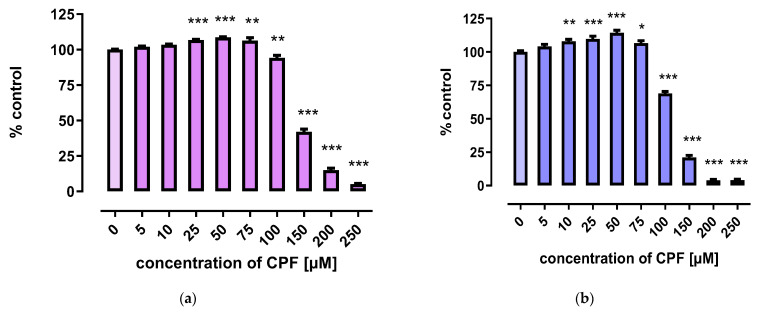
The effect of CPF on viability of 3T3-L1 cells measured by the MTT assay (**a**) and Neutral Red (NR) uptake assay (**b**) after 24 h incubation with medium (control) or chlorpyrifos (CPF). The results are expressed as a percentage of the control. Means ± SEM (standard error mean) (*n* = 12). Statistical significance of the difference from the control: * *p* < 0.05; ** *p* < 0.01; *** *p* < 0.001.

CPF slightly increased the viability of 3T3-L1 cells in low concentrations (25–75 μM). However, decreased viability was observed after treatment with concentrations exceeding 100 μM (Figure 1).

**Figure 2:**
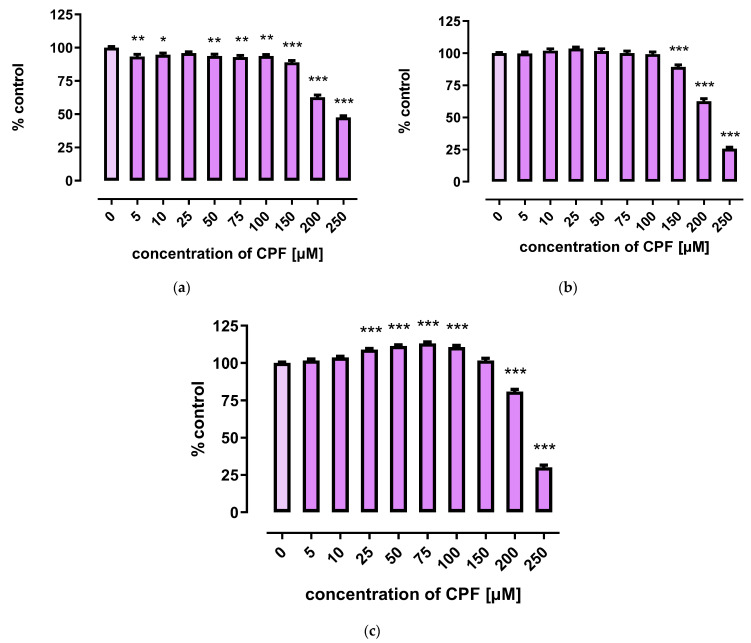
The effect of CPF on proliferation of 3T3-L1 cells measured by the MTT assay after 24 h (**a**), 48 h (**b**) and 72 h (**c**) incubation with medium (control) or chlorpyrifos (CPF). The results are expressed as a percentage of the control. Means ± SEM (standard error mean) (*n* = 12). Statistical significance of the difference from the control: * *p* < 0.05; ** *p* < 0.01; *** *p* < 0.001.

**Figure 3:**
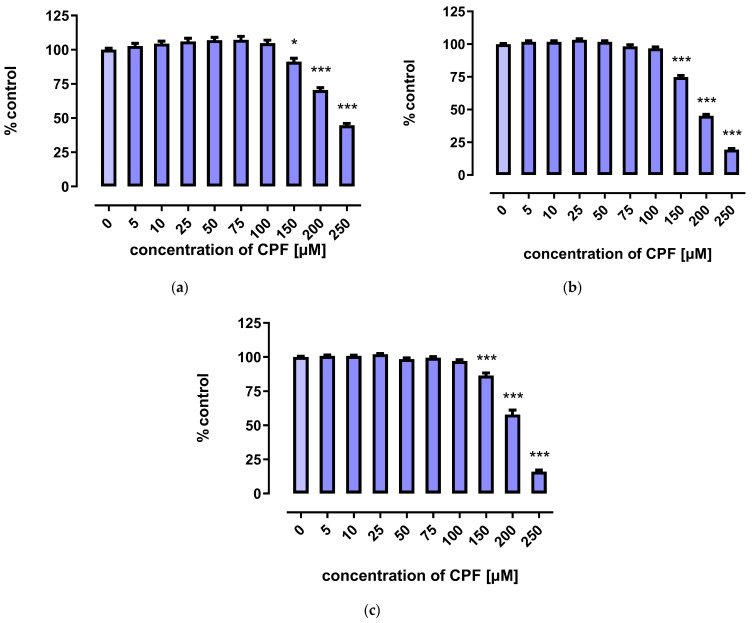
The effect of CPF on proliferation of 3T3-L1 cells measured by the Neutral Red (NR) uptake assay after 24 h (**a**), 48 h (**b**), and 72 h (**c**) incubation with medium (control) or chlorpyrifos (CPF). The results are expressed as a percentage of the control. Means ± SEM (standard error mean) (*n* = 12). Statistical significance of the difference from the control: * *p* < 0.05; *** *p* < 0.001.

**Figure 4:**
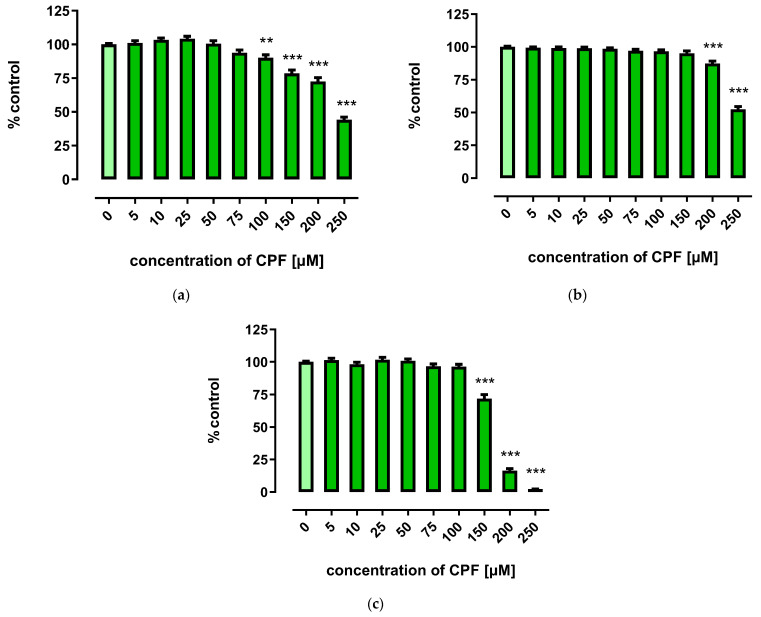
The effect of CPF on proliferation of 3T3-L1 cells measured by the BrdU assay after 24 h (**a**), 48 h (**b**), and 72 h (**c**) incubation with medium (control) or chlorpyrifos (CPF). The results are expressed as a percentage of the control. Means ± SEM (standard error mean) (*n* = 12). Statistical significance of the difference from the control: ** *p* < 0.01; *** *p* < 0.001.

A marked decrease in proliferation of CPF-treated 3T3-L1 cells was observed with concentrations exceeding 150 µM. However, the three tests used to assess cells’ proliferation provided less coherent results. Though the MTT assay showed that CPF concentrations of 25–100 μM decreased cell proliferation after 24 h of incubation, prolonged exposure time (72 h) led to an increase in cell proliferation (Figure 2). However, the remaining two assays revealed only a decrease in cell proliferation at concentrations exceeding 150 μM (Figure 3 and Figure 4).

### 2.2. Effect of CPF on Differentiation of 3T3-L1 Cells to Adipocytes

**Figure 5:**
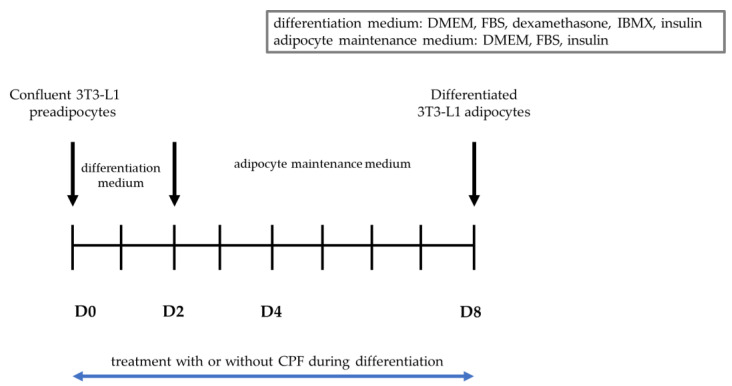
Differentiation process of 3T3-L1 cells.

Figure 5 shows a scheme indicating the differentiation process of 3T3-L1 cells (described in Section 4).

**Figure 6:**
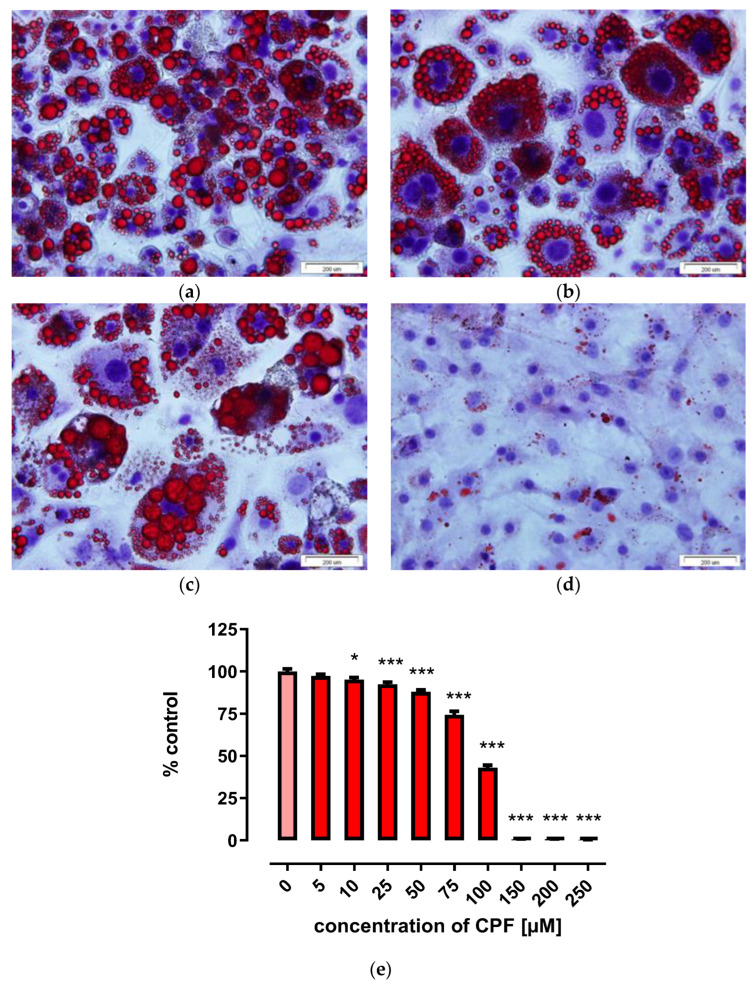
Effect of CPF on lipid droplet accumulation using Oil Red O staining in 3T3-L1 cells. On day 8 of maturation, the cells were stained with Oil Red O and hematoxylin and photographed. Control (**a**), 25 µM CPF (**b**), 75 µM CPF (**c**) and 150 µM CPF (**d**), spectrometric quantification of Oil Red O staining (**e**), see Section 4 for details. The results are expressed as a percentage of untreated control. Means ± SEM (standard error mean) (*n* = 3). Statistical significance of the difference from the control: * *p* < 0.05; *** *p* < 0.001.

Triglyceride accumulation and fatty acid uptake are characteristic features of adipocytes [34]. Triglyceride accumulation can be visualized by Oil Red O staining, as described in Materials and Methods. In our experimental design, untreated differentiated adipocytes served as a control for treated cells. Control cells showed a high degree of Oil Red O staining, indicating a high degree of lipid droplet accumulation (Figure 6a). CPF inhibited Oil Red O staining in a concentration-dependent manner (Figure 6b–d, summarized in Figure 6e, and Figure S1 in Supplementary Materials). Differentiated Oil Red O-stained cells were still observed when preadipocytes were treated with 25 µM CPF (Figure 6b) or 75 µM (Figure 6c), but no lipid droplets were observed after treatment with 150 µM CPF (Figure 6d) or higher. CPF exposure caused hypertrophy of adipocytes. Results are summarized in Figure 6e.

### 2.3. Effect of CPF on Expression of Adipogenesis Promoting Genes

**Figure 7:**
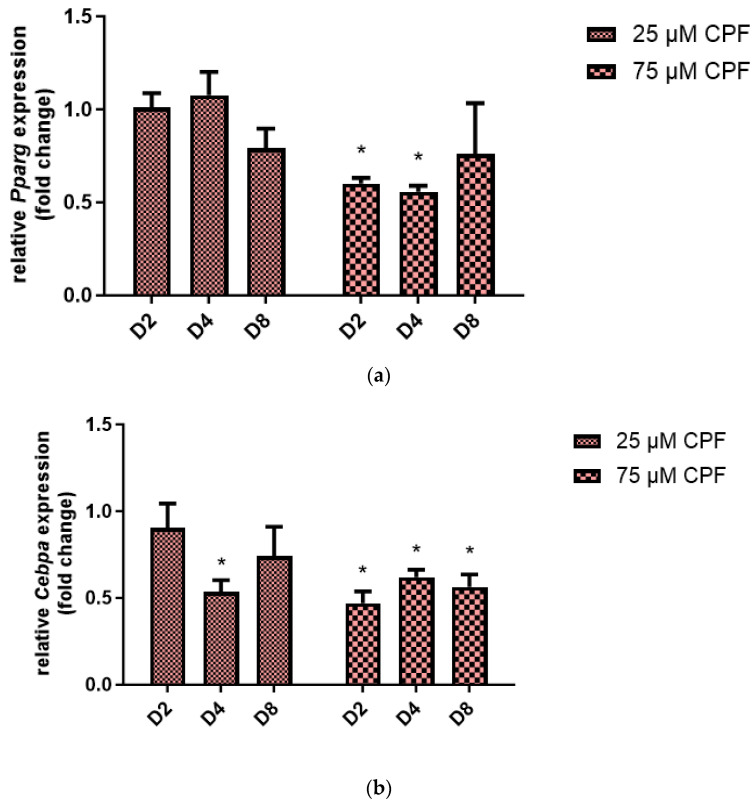
The effect of CPF on adipogenic regulatory genes expression during 3T3-L1 cells differentiation. Expression of *Pparg* mRNA (**a**) and *Cebpa* mRNA (**b**). Results are expressed as fold change of gene expression compared with the control after normalization with reference gene 18S ribosomal RNA. Mean ± SD (*n* = 3). Statistical significance of the difference from the control: * *p* < 0.05.

In order to further examine the effect of CPF exposure on the differentiation of preadipocytes to adipocytes, *Pparg* (Figure 7a) and *Cebpa* (Figure 7b) mRNA expression were examined. We measured the expression of adipogenesis regulatory genes at the end of each stage of adipogenesis: Induction (D2), differentiation (D4) and maturation (D8). Treatment with CPF at concentrations of 25 µM had no influence on *Pparg* expression during all analyzed adipogenesis stages (D2, D4, and D8). A higher concentration of CPF decreased *Pparg* expression at D2 and D4 compared with the control. Exposure to 25 µM CPF decreased *Cebpa* mRNA expression; however, the fold change was statistically significant only at D4. Treatment with 75 µM CPF caused a significant decrease in *Cebpa* expression at all analyzed adipogenesis stages (D2, D4, and D8).

### 2.4. Effect of CPF on Free Fatty Acid Uptake in Adipocytes

#### 2.4.1. Exposure of Fully Differentiated Adipocytes to CPF

**Figure 8:**
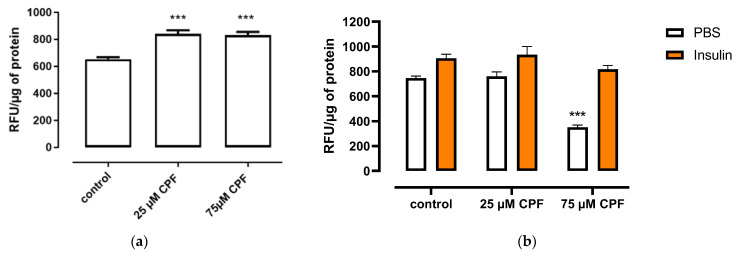
Effect of CPF on basal or insulin-stimulated fatty acid uptake by mature adipocytes. (**a**) Mature adipocytes were treated with CPF in the presence of BODIPY for 24 h. (**b**) Mature adipocytes were treated with CPF for 24 h and then stimulated with vehicle (PBS) or insulin (100 nM) in the presence of BODIPY for 60 min. Means ± SEM (standard error mean) (*n* = 3). Statistical significance of the difference from the control: *** *p* < 0.001. (**b**): PBS-stimulated control vs. CPF-treated and PBS-stimulated cells; insulin-stimulated control vs. CPF-treated and insulin-stimulated cells.

CPF exposure significantly increased basal free fatty acid uptake, compared to control, although the effect was not dose dependent (Figure 8a). However, CPF exposure had no effect on the uptake of insulin-stimulated fatty acid, compared to the control (Figure 8b).

#### 2.4.2. Exposure of 3T3-L1 Cells to CPF during Differentiation

**Figure 9:**
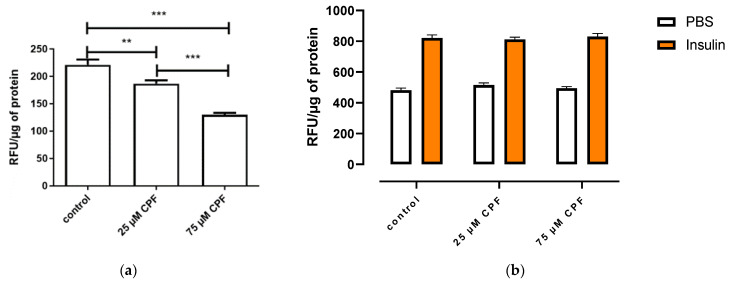
Basal (**a**) and insulin-stimulated (**b**) fatty acid uptake by mature adipocytes exposed to CPF during differentiation. (**a**) When maturated, adipocytes were treated with BODIPY for 24 h. (**b**) When maturated, adipocytes were treated with vehicle (PBS) or insulin (100 nM) in the presence of BODIPY for 60 min. Means ± SEM (standard error mean) (*n* = 3). Statistical significance of the difference from the control: ** *p* < 0.01; *** *p* < 0.001. (**b**): PBS-stimulated control vs. CPF-treated and PBS-stimulated cells; insulin-stimulated control vs. CPF-treated and insulin-stimulated cells.

3T3-L1 cells were exposed to CPF during differentiation, and then basal (Figure 9a) and insulin-stimulated (Figure 9b) free fatty acid uptake were assessed. Basal free fatty acid uptake decreased after 24 h of exposure to mature adipocytes. The effect was concentration dependent. CPF exposure had no effect on insulin-stimulated free fatty acid uptake.

## 3. Discussion

Due to the lipophilic nature of CPF, it is sequestered and accumulated in adipose tissue and may affect adipocytes and their functions. Adipocytes originate in a complicated, multi-stage process of adipocyte differentiation from primary cells called adipogenesis. The two major transcription factors in adipogenesis are peroxisome proliferator-activated receptor γ (PPARγ) and CCAT enhancer-binding protein alpha (C/EBPα) [35]. The presented study investigated the role of CPF in the proliferation and differentiation of 3T3-L1 preadipocytes into mature adipocytes. We also assessed the impact of this pesticide on the uptake of fatty acids.

The study also revealed the cytotoxic effect of CPF on 3T3-L1 cells at concentrations of 100 μM and higher. Toxicity of CPF has been demonstrated, inter alia, in the human monocyte cell line U937 [36], human T cells [37], human neuroblastoma cell line SH-SY5Y [38], C3H10T^1/2^ cells [39], lung carcinoma A549 cells [40], human peripheral blood lymphocytes, and an HepG2 cell line [41]. The MTT assay results reflecting the activity of NADH-dependent cellular oxidoreductase are coherent with in vivo studies showing reduced activities of mitochondrial enzymes after CPF exposure [42,43].

Interestingly, in our experimental design, CPF in lower concentrations increased the viability of 3T3-L1 cells. Similar results were obtained by Blanco et al. (2020) [44] on 3T3-L1 cells and by Sandhu et al. (2017) [39] on C3H10T^1/2^ cells. In vitro studies have also shown that exposure to a low concentration of CPF promotes the proliferation of MCF-7 cells, whereas higher concentrations of this pesticide lead to cell death [45]. It is possible that this effect is a consequence of adaptation to oxidative stress, as sublethal exposure to cellular stressors encourages the production and secretion of specific molecules needed to lessen damage and boost survival, enabling cells to react effectively to a higher concentration of the same stressor [46]. Induction of oxidative stress after CPF exposure has been shown in MDA-MB-231 and MCF-7 cells [47], PC12 cells [48,49], and cerebellar granule neurons [50]. It has also been shown that CPF generates reactive oxygen species in *Drosophila melanogaster* [51] and the cardiac tissue of rats [52].

As hyperplasia of adipocytes is an important process in the development of obesity, we assessed the impact of CPF on preadipocyte proliferation with different methods. Interestingly, the results of the BrdU assay and the NR assay did not reveal an increased proliferation of the cells, as demonstrated by the MTT assay. Since MTT assay measures correspond rather to the metabolic activity of adipocytes, it might be assumed that the results of all three assays taken together revealed an increase in cellular metabolism rather than an increase in the number of cells. This is in accordance with previously published data indicating normal growth of 3T3-L1 cells after CPF exposure [53]. Simultaneously, CPF exposure did not affect the proliferation of HT-29, HepG2, and THLE3 cells [54]. On the contrary, CPF inhibited the proliferation of MCF-7 and MDA-MB-231 breast cancer cells through ERK1/2 phosphorylation [47], while promoting colorectal adenocarcinoma H508 cell growth through the activation of the EGFR/ERK1/2 signaling pathway. Thus, the impact of CPF on the proliferation of cells appears to be tissue and even cell type specific.

Murine 3T3-L1 cells are one of the best-characterized and most reliable in vitro models for studying adipogenesis and preadipocyte differentiation. Adipocyte differentiation is a multi-step process requiring the involvement of multiple signaling pathways. PPARγ and C/EBPα are two key transcription factors in adipogenesis. They are involved in the regulation of adipogenesis, inducing the expression of genes that are involved in insulin sensitivity, lipogenesis, and lipolysis [55,56,57]. Research indicates that environmental chemicals and food contaminants that activate PPARγ typically induce adipogenic differentiation [58]. In our experimental design, exposure to CPF resulted in down-regulation of C/EBPα and PPARγ, as well as decreased lipid accumulation.

The discussed findings are consistent with previously published data indicating that CPF at a very low concentration (5.7–22.8 μM) did not affect the differentiation of 3T3-L1 cells; however, the authors did not publish the precise protocol of this research [53]. The antiadipogenic effect of CPF exposure on 3T3-L1 cells and NIH-3T3 has been described by Taxvig et al. [58]. Although CPF had no effect on the expression of mPPARα and mPPARγ, it decreased lipid accumulation. Similar results were obtained for the C_3_H_10_T^1/2^ cell line. Interestingly, exposure to CPF accompanied by retinoic acid resulted in adipogenic differentiation through a process involving a crosstalk at GSK3β signaling [39]. Therefore, interactions of CPF with other chemicals should be considered, as this may modify the effect resulting from exposure to this pesticide. Inhibited adipogenesis of 3T3-L1 cells was also shown after glyphosate-based pesticide exposure [59,60]. This OP inhibited the differentiation of preadipocytes when cells were treated with it during the induction of differentiation. Interestingly, removal of the glyphosate-based pesticide restored differentiation [55]. Glyphosate-based pesticides also inhibited differentiation of mouse embryonic fibroblasts, which was associated with inhibited expression of PPARγ [60].

Conversely, it has been reported that CPF and its metabolite 3,5,6-trichloropyridinol (TCP) have an obesogenic potential. These substances were shown to act by increasing the number of differentiated 3T3-L1 cells and their ability to store lipid droplets, which occur through the upregulation of transcription factors C/EBPα and PPARγ [44]. However, the experimental protocol used by Blanco et al. [44] differs from our protocol and the protocol used by Taxvig et al. [58]. Blanco et al. [44] exposed 3T3-L1 cells to CPF and TCP once at the beginning of the adipocyte differentiation process, whereas in our research, 3T3-L1 cells were treated with CPF through the whole differentiation process. Interestingly, it was shown that TCP concentration, which increased cell viability, led to a drastic reduction in lipid accumulation [44].

CPF exposure affects intracellular lipid accumulation in adipocytes and other types of cells. In 3T3-L1 differentiating preadipocytes, CPF exposure increased the accumulation of triglycerides [44]. An increase in neutral lipid accumulation was also observed in McArdle-RH7777 hepatoma cells exposed to CPF. The effect was ascribed to increased de novo lipogenesis, increased free fatty acid accumulation from extracellular sources, and decreased fatty acid-induced triglyceride secretion [61]. On the contrary, He et al. demonstrated that CPF had no effect on intracellular triglyceride accumulation in HepG2 cells [62]. Such differences may be a result of species-dependent sensitivity to CPF and/or differences in the lipogenesis machinery between these two cell types. Our findings showed that the effect of CPF exposure depends on the differentiation stage of cells. CPF increased basal free fatty acid uptake in mature adipocytes, an effect likely associated with the more intense uptake of fatty acids by adipocytes than preadipocytes. On the other hand, CPF decreased basal-free fatty acid uptake when added during the differentiation process. We speculate that the discrepancy between the effects of CPF on mature and differentiating cells may be due to the impact of CPF on adipogenesis. As described above, we demonstrated that CPF reduces the differentiation of preadipocytes to mature adipocytes. Therefore, it is likely that decreased fatty acid uptake is more due to the reduced number of fully differentiated cells than to the influence of CPF on mechanisms directly related to the uptake and accumulation of fatty acids. To the best of our knowledge, this is the first study to demonstrate the effect of OPs on adipocyte fatty acid uptake. It has been reported on NIH3T3-L1 cells that organochlorine pesticides increased fatty acid uptake in mature adipocytes and had no effect on insulin-stimulated fatty acid uptake or lipolysis [63]. The authors suggested that increased fatty acid uptake may be due to increased diffusion-mediated fatty acid uptake due to altered membrane composition [64,65]. Moreover, we suggest that exposure to organochlorine compounds may promote adipocyte hypertrophy due to increased fatty acid accumulation.

Numerous studies indicate that CPF can cause changes in the function of cells, inducing cell damage, and is therefore a potential risk factor for the health of humans and organisms in the environment. Our results showed that CPF inhibited the proliferation and differentiation of preadipocytes. Inhibition of differentiation occurred due to the reduced expression of the key adipogenesis transcription factors, C/EBPα and PPARγ. However, our results are limited by the fact that the differentiation medium contained dexamethasone. Since cells growing in dexamethasone-free medium were not included in our experimental design, it is possible that CPF acted as a factor stimulating differentiation of pre-adipocytes, but this effect was weaker than that of the adipogenic cocktail.

The obtained results also suggest that CPF leads to disturbances in adipose tissue function and may affect the development of obesity through the effect it has on fatty acid uptake and accumulation. During childhood and adolescence, obesity is mainly associated with hyperplasia, while in adulthood it is associated with hypertrophy of mature adipocytes. Our finding strongly supports the idea that CPF may lead to adipocyte hypertrophy due to increased fatty acid accumulation in fully differentiated adipocytes.

## 4. Materials and Methods

All chemicals were purchased from Merck KGaA (Darmstadt, Germany), if not indicated otherwise. CPF (O,O-diethyl O-3,5,6-trichloropyridin-2-yl phosphorothioate; CAS No. 2921-88-2; purity ≥ 98.0%) was purchased from Merck KGaA (Darmstadt, Germany). CPF stock solutions (100 mM) were prepared in dimethylsulfoxide (DMSO). Working solutions were made by dissolving an appropriate stock solution in culture medium.

### 4.1. Cell Culture and Differentiation

The 3T3-L1 mouse cell line (ATCC CL-173^™^) was cultured in Dulbecco’s Modified Eagle’s Medium (DMEM) supplemented with 10% bovine calf serum (BCS; Biological Industries Israel Beit-Haemek), glutamine (4 mM), 100 U/mL penicillin, and 100 mg/mL streptomycin. Cells were maintained in a humidified atmosphere of 95% air and 5% CO_2_ at 37 °C. Preadipocytes were passaged when they were approximately 70–80% confluent. 3T3-L1 preadipocytes were differentiated according to the ATCC^®®^ protocol [66]. To initiate differentiation into mature adipocytes, preadipocytes were allowed to grow for 48 h, or until they reached 100% confluency, and were then incubated as a confluent culture for another 48 h. Two days after reaching confluency (D0), the media was removed and replaced by differentiation medium containing 90% DMEM, 10% fetal bovine serum (FBS), glutamine (4 mM), 100 U/mL penicillin, 100 mg/mL streptomycin, 1 µM dexamethasone, 0.5 mM methylisobutylxanthine (IBMX), and 1 µg/mL insulin. After 48 h (D2), the differentiation medium was removed and replaced by adipocyte maintenance medium containing DMEM supplemented with 10% FBS, glutamine (4 mM), 100 U/mL penicillin, 100 mg/mL streptomycin, and 1 µg/mL insulin. Adipocyte maintenance medium was replaced every 48 to 72 h.

### 4.2. Cell Viability and Proliferation Assessment—MTT Assay

The sensitivity of 3T3-L1 cells to CPF activity was evaluated using a standard spectrophotometric 3-(4,5-dimethylthiazole-2-yl)-2,5-diphenyltetrazolium bromide (MTT) assay. Cells were seeded on 96-well plates at a density of 5 × 10^3^ cells/well for toxicity tests or 1 × 10^3^ cells/well for assessment of proliferation. After 24 h of incubation, the medium was replaced by the fresh one, which contained 2% BCS (for toxicity tests) or 10% BCS (for cell proliferation tests) and appropriate concentrations of CPF (5, 10, 25, 50, 75, 100, 150, 200, 250 µM). After 24 h incubation in the case of toxicity testing, and after 24, 48, or 72 h incubation in the case of proliferation assessment, MTT solution (5 mg/mL; 15 µL/well) was added and incubated for 3 h at 37 °C in a humidified atmosphere of 95% air and 5% CO_2_. Purple crystals of formazan were solubilized in 100 µL/well DMSO. The absorbance was measured at 570 nm wavelength using an Omega FLUOstar Microplate Reader (BMG LABTECH, Ortenberg, Germany).

### 4.3. Cell Viability and Proliferation Assessment—NR Assay

3T3-L1 cells were seeded on 96-well plates at a density of 5 × 10^3^ cells/well (for toxicity tests) or 1 × 10^3^ cells/well (for cell proliferation tests). After 24 h of incubation, the medium was replaced with the fresh one, which contained 2% BCS (for toxicity tests) or 10% BCS (for cell proliferation tests) and appropriate concentrations of CPF (5, 10, 25, 50, 75, 100, 150, 200, and 250 µM). After 24 h of incubation in the case of the toxicity test, and after 24, 48, or 72 h incubation in the case of proliferation assessment, the medium was discarded and neutral red (NR) solution (40 µg/mL; 100 µL/well) was added and incubated for 3 h at 37 °C in a humidified atmosphere of 95% air and 5% CO_2_. After incubation, the medium was removed and the cells fixed with 1% CaCl_2_ in 0.5% paraformaldehyde. Subsequently, the incorporated dye was solubilized using 1% acetic acetate in a 50% ethanol solution. The absorbance was measured at 540 nm wavelength using the Omega FLUOstar Microplate Reader (BMG LABTECH, Germany).

### 4.4. Cell Proliferation—BrdU Assay

Cell proliferation was evaluated by a bromodeoxyuridine (BrdU) cell proliferation ELISA (Roche, Mannheim, Germany). 3T3-L1 cells were seeded on 96-well plates at a density of 2 × 10^3^ cells/well. After 24 h of incubation, the medium was replaced by the fresh one alone (control) or with indicated concentrations of CPF (5, 10, 25, 50, 75, 100, 150, 200, 250 µM). After 24, 48, or 72 h incubation, BrdU was added, and the following steps were performed according to the manufacturer’s protocol. Absorbance was measured at 450 nm wavelength using the Omega FLUOstar Microplate Reader (BMG LABTECH, Germany).

### 4.5. Oil Red O Staining

3T3-L1 cells were seeded in a 24-well plate and differentiated into mature adipocytes according to the previously described procedure. Vehicle control or CPF (5, 10, 25, 50, 75, 100, 150, 200, 250 µM) was added with differentiation medium and adipocyte maintenance medium. On day 8, cells were washed with phosphate-buffered saline (PBS) and fixed with 10% paraformaldehyde for 60 min. The cells were then washed twice with distilled water and rinsed with 60% isopropanol for 2–5 min. Next, cells were stained for 20 min with a filtered Oil Red O solution (1.8 mg/mL in isopropanol:water 3:2). Stained cells were washed four times with distilled water and incubated with 100% isopropanol for 10 min to extract Oil Red O from the cells. Absorbance was measured at 510 nm wavelength using the Omega FLUOstar Microplate Reader (BMG LABTECH, Germany).

Oil Red O staining was also used to visualize cytosolic lipids. 3T3-L1 cells were seeded in Petri dishes and differentiated into mature adipocytes. Vehicle control or CPF (5, 10, 25, 50, 75, 100, 150, 200, 250 µM) was added with differentiation medium and adipocyte maintenance medium. On day 8, cells were washed with PBS and fixed with 10% paraformaldehyde for 60 min. The cells were then washed twice with distilled water and rinsed with 60% isopropanol for 2–5 min. Subsequently, cells were stained for 20 min with a filtered Oil Red O solution (1.8 mg/mL in isopropanol:water 3:2). Stained cells were washed four times with distilled water, and then the nuclei were stained with hematoxylin for 1 min. After being rinsed with distilled water, stained cells were observed with the Olympus BX51 (Olympus, Hamburg, Germany) imaging system with 20×/40× objectives.

### 4.6. Isolation of Total RNA

3T3-L1 cells were seeded in a 6-well plate and differentiated into mature adipocytes according to the previously described procedure. Vehicle control or CPF (25 and 75 µM) was added with differentiation medium and adipocyte maintenance medium. At D0, D2, D4, and D8, the cells were washed with PBS, and the total RNA was isolated using the High Pure RNA Isolation Kit (Roche, Mannheim, Germany) according to the manufacturer’s protocol. The RNA quality and quantity were measured using NanoDrop^®®^ 1000 (Thermo Fisher Scientific, Waltham, MA, USA) by the A260/A280 absorbance ratio.

### 4.7. Real-Time RT-PCR Quantification of Gene Expression

cDNA was generated from 2 µg of total RNA by using the High Capacity cDNA Reverse Transcription Kit (Applied Biosystems^®®^, Thermo Fisher Scientific, Waltham, MA, USA) in a 20 µL reaction mixture. Two microliters of cDNA solution was used to amplify cDNA using TaqMan^®®^ Fast Universal PCR Master Mix (2×) and TaqMan^®®^ Gene Expression Assays (PPAR-gamma: Mm00440940_m1; C/EBP alpha: Mm00514283_s1; Rn18s: Mm03928990_g1), according to the manufacturer’s protocol (Applied Biosystems^®®^; Thermo Fisher Scientific, Waltham, MA, USA). Real-time RT-PCR was performed in an ABI 7500 Fast RealTime PCR System (Applied Biosystems^®®^, Thermo Fisher Scientific, Waltham, MA, USA). The 18S ribosomal RNA gene was used as a control to normalize the values by the 2(−ΔΔCt) method (Livak and Schmittgen, 2001).

### 4.8. Fatty Acid Uptake

The effect of CPF exposure on fatty acid uptake was determined in mature (D12) 3T3-L1 adipocytes with the fluorescent dye 4,4-difluoro-5-methyl-4-bora-3a,4a-diaza-s-indacene-3-dodecanoic acid (BODIPY 500/510 C1, C12; Thermo Fisher Scientific, Waltham, MA, USA). 3T3-L1 cells were seeded in a 96-well black plate with clear flat bottoms and differentiated into mature adipocytes according to the previously described procedure. On day 12 (D12), DMEM containing 10% FBS was removed, and cells were incubated in DMEM containing 0.2% BSA for 6 h for acclimation. After acclimation, cells were treated with DMEM containing 0.2% fatty acid-free BSA and 2.5 μM of BODIPY with vehicle or CPF (25 or 75 μM) for 24 h to determine basal fatty acid uptake. To assess whether exposure to CPF influenced insulin-stimulated fatty acid uptake, cells were treated for 24 h with vehicle or CPF (25 or 75 μM) in DMEM containing 0.2% BSA following a 6 h acclimation period. After that, adipocytes were washed and incubated in DMEM containing 0.2% BSA, BODIPY (2.5 μM), and insulin (100 nM) or vehicle (PBS) for 60 min. After either 24 h of exposure or 1 h of insulin stimulation, the cells were washed three times with PBS and incubated with lysis buffer (0.2% SDS) overnight. Fluorescent intensity within the cell lysates was determined at an excitation of 485 nm and emission of 520 nm using the Omega FLUOstar Microplate Reader (BMG LABTECH, Germany). The protein concentration was then measured using Pierce™ BCA Protein Assay Kit (Thermo Fisher Scientific, Waltham, MA, USA) according to the manufacturer’s protocol. Absorbance was measured at 562 nm wavelength using the Omega FLUOstar Microplate Reader (BMG LABTECH, Germany). Results were shown as RFU (relative fluorescence units) to µg protein ratio (RFU/µg protein).

Fatty acid uptake was also assessed in mature adipocytes exposed to CPF during differentiation. 3T3-L1 cells were differentiated into mature adipocytes according to the previously described procedure. Vehicle control or CPF (25 and 75 µM) was added with differentiation medium (D0) and adipocyte maintenance medium (D2, D5, D7, and D9). On day 12, DMEM containing 10% FBS was removed, and cells were incubated in DMEM containing 0.2% BSA for 6 h for acclimation. After 6 h, cells were treated with DMEM containing 0.2% fatty acid-free BSA and 2.5 μM of BODIPY for 24 h to determine basal fatty acid uptake. To assess whether exposure to CPF influenced insulin-stimulated fatty acid uptake, cells were treated for 24 h with DMEM containing 0.2% BSA following a 6 h acclimation period. After that, adipocytes were washed and incubated in DMEM containing 0.2% BSA, BODIPY (2.5 μM), and insulin (100 nM) or vehicle (PBS) for 60 min. After either 24 h of exposure or 1 h of insulin stimulation, the cells were washed three times with PBS and incubated with lysis buffer (0.2% SDS) overnight. Fluorescent intensity within the cell lysates was determined at an excitation of 485 nm and emission of 520 nm using the Omega FLUOstar Microplate Reader (BMG LABTECH, Germany). The protein concentration was then measured using Pierce™ BCA Protein Assay Kit (Thermo Fisher Scientific, Waltham, MA, USA) according to the manufacturer’s protocol. Absorbance was measured at 562 nm wavelength using the Omega FLUOstar Microplate Reader (BMG LABTECH, Germany). Results were shown as RFU to µg protein ratio (RFU/µg protein).

### 4.9. Statistical Analysis

Statistical analysis was performed using GraphPad Prism 5.0 software (GraphPad Software Inc., San Diego, CA, USA). Multiple comparisons were performed using one-way analysis of variance (ANOVA), followed by Dunnett’s or Tukey’s post hoc test. Data are presented as the mean ± standard error mean. *p* < 0.05 was considered statistically significant.

## Data Availability

The datasets generated during and/or analyzed during the current study are available from the corresponding author upon reasonable request.

## Supplementary Materials

The following supporting information can be downloaded at: https://www.mdpi.com/article/10.3390/ijms242216038/s1.
