# Supplementary material for: Exposure to Chlorpyrifos Alters Proliferation, Differentiation and Fatty Acid Uptake in 3T3-L1 Cells
Source: Int J Mol Sci. 2023 Nov 7;24(22):16038. doi: 10.3390/ijms242216038 (PMC10671786; doi:10.3390/ijms242216038)
Supplement: Supplementary file 1 [file ijms-24-16038-s001.zip › ijms-2654838-supplementary.pdf]

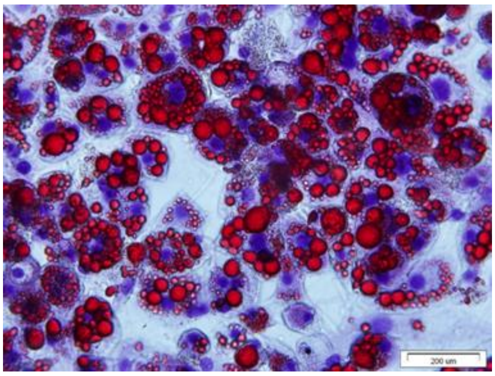

(**a**)

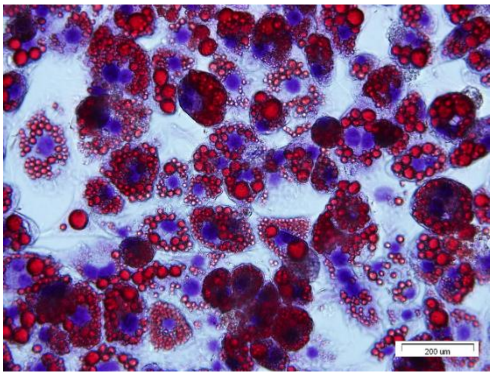

(**b**)

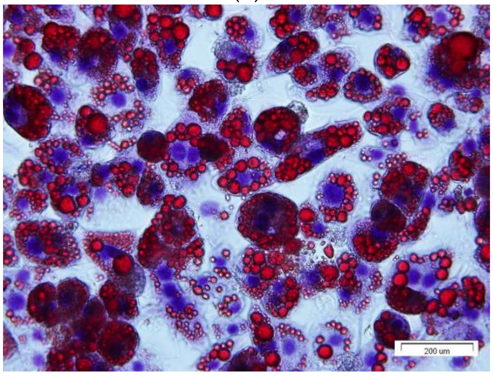

(**c**)

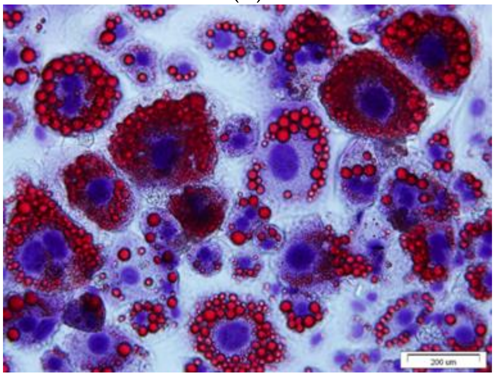

(**d**)

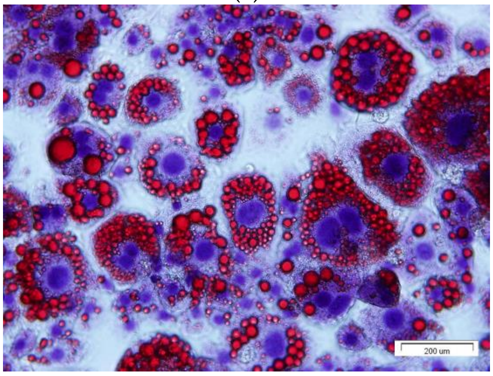

(**e**)

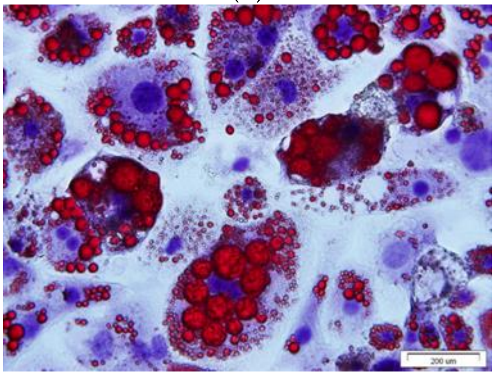

(**f**)

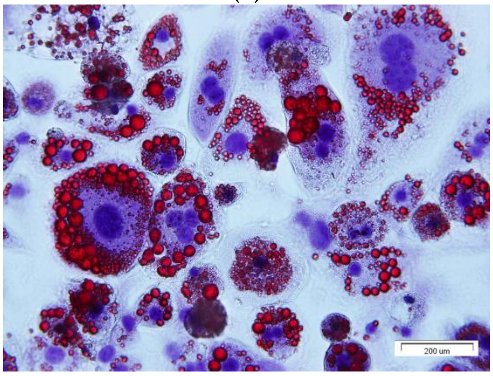

(**g**)

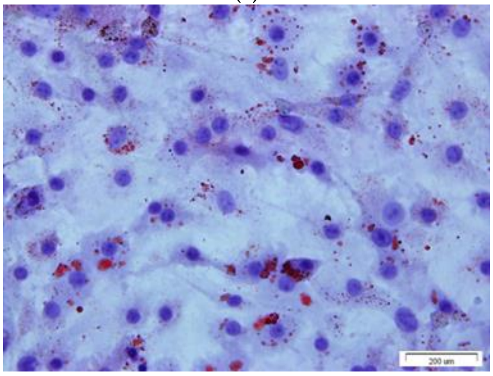

(**h**)

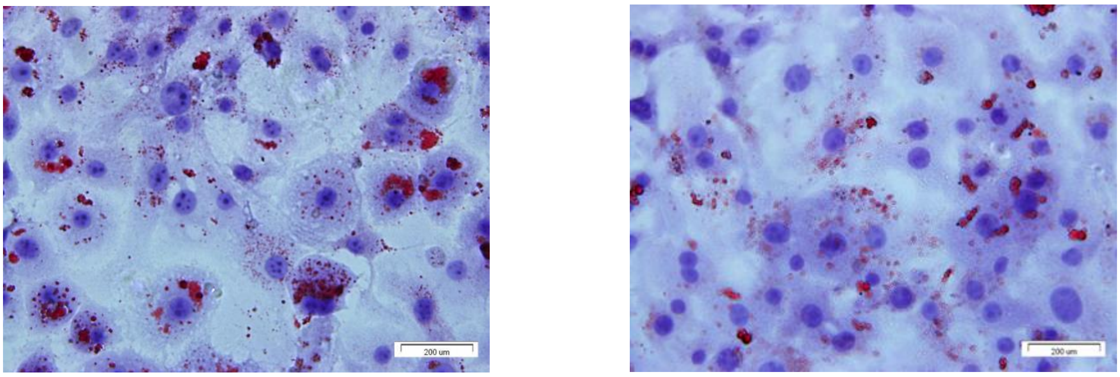

(**i**) (**j**)

Figure S1. Effect of CPF on the lipid droplets accumulation using Oil Red O staining in 3T3-L1 cells. On day 8 of maturation, the cells were stained with Oil Red O and hematoxylin, and photographed. Control **(a)**, 5 μM CPF **(b)**, 10 μM CPF **(c)**, 25 μM CPF **(d)**, 50 μM CPF **(e)**, 75 μM CPF **(f),** 100 μM CPF **(g)**, 150 μM CPF **(h)**, 200 μM CPF **(i)** and 250 μM CPF **(j)**, see Materials and Methods for details**.**
